# Study on the Effect of Microwave Processing on Asphalt-Rubber

**DOI:** 10.3390/ma13020411

**Published:** 2020-01-15

**Authors:** Jing Xu, Rui Li, Tao Liu, Jianzhong Pei, Yongkang Li, Qinghui Luo

**Affiliations:** 1 School of Highway, Chang’an University, Xi’an 710064, China; jingxv@chd.edu.cn (J.X.); liutao@chd.edu.cn (T.L.); peijianzhong@126.com (J.P.); liyongkang@chd.edu.cn (Y.L.); 2 Chongqing Municipal Research Institute of Design, Chongqing 400000, China; Luoqh_chd@163.com

**Keywords:** asphalt-rubber, microwave processing, swelling, dissolution, viscosity, high-temperature performance, low-temperature performance

## Abstract

The addition of crumb rubber (CR) into base asphalt plays a critical role in the improvement of the performance of Asphalt-Rubber (AR) binders. However, due to the problems, like high constructing temperature and energy consumption brought by the additional rubber, the use of AR binders could be limited in some areas. During this study, CR is processed by microwave is adopted to reduce the viscosity of the AR binders system, while the CR processed by long screw extrusion also is studied. First, the swelling (the absorption of light component into the CR particle) and dissolution (some molecules of CR dissolving into the base asphalt), both of which determine the improved performance of AR binders, are investigated by fluorescence microscopy and extraction tests. The size of the CR particle after swelling observed by fluorescence microscopy is used to evaluate the swelling rate of CR samples, and the ratio of the weight loss of CR samples after extraction to the original weight is employed to measure the dissolution rate. Then, Brookfield rotational viscometer and storage stability tests are conducted. Last, the rheologic performance, including high and low-temperature performances, is characterized by the dynamic shear rheometer (DSR) and bending beam rheometer (BBR), respectively. The fluorescence microscopy and extraction results show that microwave processing could effectively increase the swelling and dissolving rate, with the figures rising twofold and more than threefold, respectively. The results show that microwave processing could effectively reduce the viscosity of AR binders, with a viscosity decrease of 65% at 190 °C and, at the same time, the high temperature of Performance Grade (PG) decrease from 88 °C to 76 °C. The storage stability could be negatively impacted, but it is slight and the low-temperature performance is improved slightly.

## 1. Introduction

Asphalt Rubber (AR) generally refers to the mixture containing crumb rubber (CR), which is obtained by grinding the recycled crumbs of tires with base asphalt [1]. Compared to just the base asphalt, AR not only has better resistance to high-temperature permanent deformation, low-temperature deformability, anti-ageing and anti-fatigue properties [2,3,4,5,6,7,8], but has an obvious noise reduction property due to CR’s high damping property [9]. So, if AR could be used worldwide, it not only could act as a way to improve the performance of pavement, but it could be a solution to recycle the great number of waste tires, providing high returns including both environmental protection and financial aspects. 

The improvement of the performance of the AR binders is thought to be mainly coming from the adding of CR [10,11]. Actually, this improvement lies in two key effects that the CR brings about: the particle effect (PE) and interaction effect (IE) between CR and asphalt [12]. PE refers to the CR particles acting as fillers in the base asphalt, reinforcing the AR system [8]. IE mainly comes from swelling (absorption of the light components from the base asphalt to CR), molecule dissolution (rubber molecules dissolving into the asphalt) and some physical interactions between rubber and asphalt [2,13,14]. Putman, Amirkhanian, et al. identified the IE and PE in AR binders, and quantified them with Equations (1) and (2) [10,12,15]. Subsequently, Singh et al. employed this concept to analyze the effects PE and IE have on the rheology and fatigue properties of AR binders, respectively [8].

(1) $$\mathrm{PE}=\frac{\mathrm{Undrained}-\mathrm{Drained}}{\mathrm{Base}}$$

(2) $$\mathrm{IE}=\frac{\mathrm{Drained}-\mathrm{Base}}{\mathrm{Base}}$$

The swelling of CR could enhance both PE and IE, since it has the light absorbance component of asphalt, one of the main parts of forming IE, and increases the volume of CR particles, which has a positive impact on PE [9,14]. Additionally, the reduced light components in the base asphalt caused by absorption and the increased volume of CR particles make the movement of CR particles difficult, both of which would increase the viscosity of binders, leading to an increase of thickness of the film coating the aggregates, which in turn, has a positive impact on the anti-fatigue and anti-ageing properties of AR pavement [11,16,17,18,19,20,21]. Additionally, the swelling rate is also decided by the nature of the CR and the asphalt chemical component [2,14,22]. Generally, desulphurization rubber has a higher swelling rate as do smaller CR, while the bigger CR has a lower rate due to its bigger surface area contacting the base asphalt [22,23,24]. Meanwhile, desulphurization by microwave treatment on crumb rubber has proven that it could benefit the crumb rubber-modified asphalt in many aspects, including viscoelastic nature, storage stability and low-temperature performance [25]. Regarding the base asphalt, research has shown that CR swelled quicker in the base asphalt containing more of the light component (Aromatic and Saturation) [2,13].

The preparation also could be an important process which decides whether the expected performance of AR binders can be obtained or not and, during this process, the main concerning factors include mixing types, time and temperature [26,27,28,29]. AR binders mixed by a high-speed shear mixer performed better at low temperature while AR binders processed by a propeller-type mixer (low-speed mixer) had better intermediate and high-temperature performances and, also, were more resistant to fatigue cracking [29]. The longer mixing time and temperature could enable more IE and PE get established. Actually, compared with mixing temperature, the effect of mixing time could be limited, especially when the time exceeds 60 minutes, while a higher mixing temperature could increase complex shear modulus, elastic recovery, and ductility [26,30,31]. Briefly, the expected performance of AR binders could be obtained by reasonably controlling mixing parameters.

AR binders with great performance, however, are strictly used in some areas due to the extremely unpleasant odor and high constructing temperature caused by high viscosity. To reduce the viscosity of AR binders, the microwave is adopted in this study to process the CR without seriously influencing other properties. The main objectives of this paper are as follows:

(1) To measure the effects of microwave processing on CR swelling and dissolution;
(2) To evaluate the viscosity and storage stability of AR binders after microwave processing;
(3) To study the rheologic properties of AR binders after microwave processing.

## 2. Materials and Methods 

### 2.1. Materials, Microwave Processing Method and Preparation of AR Binders

#### 2.1.1. Materials

During this study, one neat asphalt binder (SK90) was used, sourced from Korea, and was labeled as 90# based on penetration grades. Three crumb rubbers were utilized: the first came from Hebei province, China, presented as CR-1; the second sourced from Shanghai city and was processed by long screw extrusion in factory for desulphurization, named as CR-2; the last was obtained by microwave-processing CR-1, reported as CR-3. Correspondingly, the AR binders composed by these CR were named as AR-1, AR-2, and AR-3. The size of all three types of CR was mesh #40. The basic information of the base asphalt is shown in Table 1.

#### 2.1.2. The Microwave Processing 

The method that utilizes microwaves to process the CR was to desulphurize it so the viscosity of the AR binders could be reduced. The main procedure follows. Some CR was put into the oven at 60 °C for 60 min to dry the CR; about 60 grams of the CR was then sent to the microwave set at maximum power during desulphurization for 3 min. The Galanz G80F20CN2L-BB microwave was used in this study had a maximum power of 850 W and a frequency of 2450 MHZ, taking into consideration that the processing time should not be too long to prevent ageing and degradation of CR. 

#### 2.1.3. Preparation of Asphalt-Rubber Binders

During this study, all AR binders were prepared by the following procedures. First, about 500 grams of SK90# was sent into the steel container for oil bath heating to 170 °C; next, 20% by the weight of the base asphalt of CR was put into the base asphalt, slowly mixing at 100 r/min until all CR was added before the mixing speed was increased to 500 r/min; last, the mixtures were mixed for about 90 min at 180 °C and, after that, the end product AR was finally produced. 

### 2.2. Fluorescence Microscope and Extraction Experiments

#### 2.2.1. Fluorescence Microscope Scanning 

**Figure 1:**
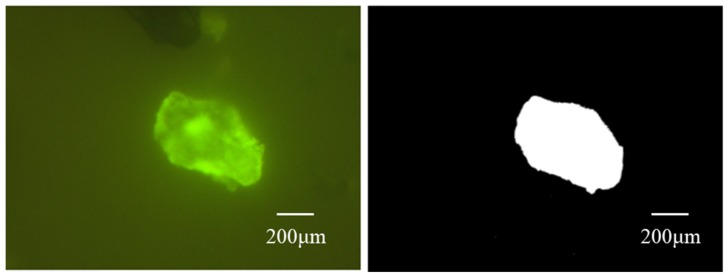
The typical example of a Fluorescence microscope scanning image (left) and the image processed by MATLAB (right).

During this study, the fluorescence microscope was used to measure the size of CR particles after swelling with the magnification of 200×, the size of which after swelling was regarded as the indicator to evaluate the swelling ability of the CR particles. The volume of CR particles was not measured due to the difficulty of finding the same CR particle before and after swelling. The typical scanning picture by fluorescence microscope is shown in Figure 1. The scanning pictures were processed by MATLAB (2016a, MathWorks, MA, USA), and the ratio of the area of the CR particles to the observation zone was calculated. Because the swelling time and the area of observation zone remained the same throughout all scanning tests, the ratio of the area of the CR particles to the observation zone could be used to characterize the swelling ability of the CR (swelling rate). This ratio was equal to the ratio of the area of the white to the black in the images below processed by MATLAB.

#### 2.2.2. Extraction Tests

To measure the mass loss of CR, which dissolve into the base asphalt, the Soxhlet extraction method with toluene as solvent was utilized. The main testing procedures were as follows. First, about 2 grams of AR binders and a filter paper were weighed and named as m_1_ and m_2_ before the binders were tightly wrapped by the filter paper; next, about 200 mL toluene was poured into the extractor after which the extractor was assembled quickly in case of volatilizing of the toluene; then, the extractor was heated until the toluene in the extractor turned colorless and, subsequently, the AR binders wrapped by filter paper were removed and the AR binders after extraction were weighed and labelled as m_3_; last, the testing was repeated while the AR binders were replaced with CR and named as m_4_, which was to obtain the dissolving rate (the mass loss) of CR into toluene. The dissolving rates of CR into the base asphalt and into toluene were named as W and W_0_, respectively, both of which were calculated according to Equations (3) and (4).

(3) $$W_{0}=\frac{m_{4}+m_{2}-m_{3}}{m_{1}}$$

(4) $$W=\frac{m_{1}\times \varepsilon +m_{2}-m_{3}}{m_{1}\times \varepsilon }-W_{0}$$

where *ε* represents the crumb rubber dosage in the Asphalt-Rubber (AR).

### 2.3. Viscosity and Storage Stability Experiments 

#### 2.3.1. Viscosity Tests

An NDJ-1C Brookfield rotational viscometer was employed to obtain the rotational viscosity of binders at 130 °C, 150 °C, 170 °C and 190 °C, respectively, following the JTG E20-2011 T0625-2011, Standard Test Method of Bitumen and Bituminous Mixtures for Highway Engineering (China).

#### 2.3.2. Stability Tests

Practically, storage stability is an important property which determines whether the great performance of binders can be obtained. The separation test was used to evaluate the storage stability of AR binders based on ASTM D7173. During the test, about 50 ± 0.5 grams of AR binders were put in an aluminum tube with the tube kept at 163 °C for 48 h, after which the tube was moved out and the softening point difference between the AR binders at the top and bottom of the tube was used to indicate the storage stability. 

### 2.4. DSR and BBR Experiments 

#### 2.4.1. Temperature Sweep Tests

A Dynamic Shear Rheometer (Anton Paar MCR 102, FL, USA) was employed to measure rheologic properties. The temperature sweep tests were conducted at the temperature range of 30–80 °C and data including the complex modulus, phase angle and rutting factor were obtained following the AASHTO T315-06. The applied strain was 10% with the frequency of 10 rad/s, and 25 mm parallel plate geometry was used during the testing. Also, the high-temperature grading could be determined based on results of rutting factors.

#### 2.4.2. Multiple Stress Creep and Recovery Tests

Multiple stress creep and recovery (MSCR) tests are utilized to evaluate the high-temperature resistance to rutting of asphalt binders, following AASTO T350. During a standard MSCR test, it includes two parts with the applied force of 0.1 kPa and 3.2 kPa, respectively. The first part contained 20 load cycles and the second part had 10 load cycles. During every load cycle there was 1 s for applying force and 9 s for resting, which simulated the vehicle load being applied on the pavement. After testing, parameters of the recoverable rate (R) and non-recoverable compliance (Jnr) were calculated according to Equations (5) and (6). Also, research showed that Jnr was well related to the performance of anti-rutting at high temperature.

(5) $$R=\frac{\gamma_{p}-\gamma_{n}}{\gamma_{p}-\gamma_{0}}$$

(6) $$\mathrm{Jnr}=\frac{\gamma_{p}-\gamma_{n}}{\tau }$$

where $\gamma_{p}$ represents the peak strain after 1 s loading of each load cycle, meanwhile $\gamma_{n}$ and $\gamma_{0}$ are the strain after 9 s resting and the strain at the beginning of each loading cycle; *τ* is the applied creep load (0.1 and 3.2 kPa). 

#### 2.4.3. BBR Tests 

Bending beam rheometer (BBR) was applied to characterize the low-temperature properties of asphalt binders, and the results of stiffness and m-value were calculated following ASTM standard D6648-08. To prepare the samples for BBR testing, the asphalt binders were heated to 160–180 °C to obtain enough mobility of the binders, then the hot asphalt binders were poured into the mold. Subsequently, the mold was put into cold water for 10 min before being demolded; last, the sample for testing was kept in a methanol bath at testing temperature for about 60 min. All samples were tested under −12 °C, −18 °C and −24 °C temperatures and the stiffness and m-value were calculated on a load time of 60 s.

## 3. Results and Discussions 

### 3.1. The Effects of Microwave Processing on the Swelling and Dissolution of CR 

The volume of CR particles after the same swelling time and under the same experimental process was adopted during this study to evaluate the swelling rate of different types of CR. The reason for adopting the volume of CR particles after swelling is the difficultly in finding and observing the same CR particle before and after swelling. Meanwhile, the initial size of CR-1, CR-2 and CR-3 were in same level (mesh #40). During the test, a fluorescence microscope at the mode of magnification of 200× was used to observe the samples, and the single swelling particle in the observation area was chosen to calculate the volume of particles. The swelling rate of different CR were evaluated by the average of ten observation values. The results of the swelling rate are shown in Table 2.

It is clear from Table 2 that the volume of CR-3 was higher than that of CR-1 and CR-2. The average value for CR-3 was about two times as high as CR-1’s, indicating that the desulphurization by microwaving has positive impacts on the swelling progress of CR. However, the figure for CR-2 was slightly lower than CR-1, although it had been desulphurized by manufacturer. The increasing particle volume could reduce the distance among particles, and swelling of CR would absorb the light component in the base asphalt, both of which would lead to the increase of viscosity of the AR binders system, but the break of the C–S and S–S bonds by microwave desulphurization would lead to the decreased viscosity. Therefore, the viscosity of AR binders was hard to judge only based on swelling results so the viscosity test would be adopted later. 

Based on the extraction experiments, both the CR dissolution rates in the base asphalt and in toluene could be obtained. It should be noted that the mass loss of AR after extracting includes molecules dissolving in asphalt and toluene, so the CR dissolution rate in the base asphalt is equal to the difference value of the mass loss rate and CR dissolution rate in toluene. Both CR dissolution rates in toluene and in the base asphalt results are shown in Table 3.

The CR dissolution rate in toluene was about the same level no matter whether the CR was desulphurized or not. This is mainly because the solvent extractive and natural rubber of CR are the two main substances that dissolve in toluene so the desulphurization process would not have an obvious change on the content of these two substances in CR. However, for dissolution rates in the base asphalt, the desulphurization would have a significant effect on this figure, with it rising more than threefold and twofold after microwave and long screw extrusion processing, respectively, compared with CR-1. Thus, it can be concluded that the desulphurization could increase dissolution rates in the base asphalt, which is great for IE. To conclude, the desulphurization could enhance both the absorption of the oily substance of the base asphalt and the molecules of CR dissolving into the base asphalt. The substance exchange between CR and the base asphalt can be accelerated by desulphurization and, as a result, the PE and IE can be established more quickly.

### 3.2. The Fundamental Properties and Storage Stability

#### 3.2.1. The Fundamental Properties 

The fundamental properties, including softening point, ductility, penetration, penetration index and elastic recovery, can be used to characterize AR binders preliminarily. The results are presented in Table 4. The SBS modified asphalt binder (SBSMA) acts as a control group.

It is clear from Table 4 that the penetration index of CR-2 (0.19) was significantly lower than CR-1 and CR-3, indicating that CR-2 was more sensitive to the temperature. The penetration of CR-1 and CR-3 was close, which demonstrates the microwave processing did not have an obvious effect on the property of temperature sensitivity. Taken from the results of the softening point, it could be known that CR-2 also had a slightly inferior performance at high temperature and it should be noted that the ductility cannot reflect the low-temperature performance well due to the particle effect of CR. Regarding the elastic recovery, samples performed closely except the figure for AR-3 was relatively lower than others.

#### 3.2.2. Storage Stability

Storage stability of modified asphalt is an issue that always concerns both the researchers and constructors. The modified asphalt with poor storage stability cannot reach the expected performance when kept in high-temperatures for a relatively long time. Concerning polymer and the organically modified asphalt, poor storage stability can lead to modifier segregation, which could cause degradation of modifiers in an accelerated way. Many measures, like heating, high-speed shearing, adding compatibilizer and so on, are adopted to improve the compatibility between modifiers and the base asphalt [32]. During this study, the difference in softening points between top and bottom was employed to evaluate storage stability, the results of which are shown in Table 5. 

The difference value of SBSMA, as a control group, was much higher than the others’, reaching more than 10 °C. During the heating process, the skin-like structures may be found in the surface, indicating the SBS modifiers segregate and float upward to the surface. All the AR samples have comparatively better storage stability. The figure for CR-2 was highest (5.9 °C), while the figures for CR-1 and CR-3 were only 3.1°C and 4.5 °C, respectively. It can be concluded that the storage stability of AR binders was not greatly influenced by microwave processing. It also should be noted that the softening points of AR in the bottom are bigger than those in the top, showing CR has a trend to sink downward.

### 3.3. Rheological Performance

#### 3.3.1. Viscosity

**Figure 2:**
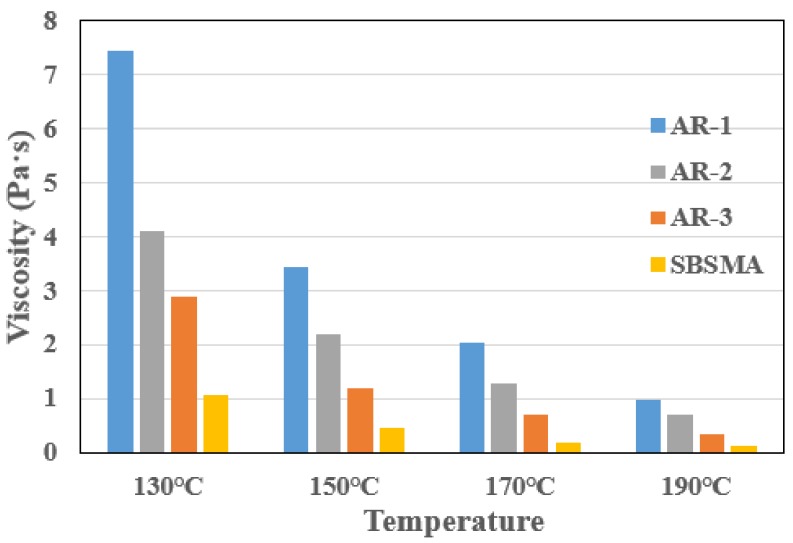
Results of Viscosity at 130 °C, 150 °C, 170 °C and 190 °C.

The viscosity of binders could not be too high during mixing and compacting processes, because higher viscosity means more energy needed to heat and obtain enough mobility and workability of the binder. So, to reduce the viscosity of AR, the microwave desulphurization process was adopted. The results of viscosity changing with temperature are presented in Figure 2.

It is clear that the viscosity of all samples decreased with the temperature, and the viscosity of CR-3 was significantly lower than CR-1 and CR-2 at all temperatures. Meanwhile CR-2 also was consistently lower than CR-1. The figures for all AR binders, however, were still higher than SBSMA. Concerning how many mixing and compacting temperatures could be reduced after microwave processing, combination of the reduced viscosity and practical use in constructing still is required. It can be concluded that the desulphurization on CR could effectively reduce the viscosity of AR binders, especially using microwave processing. This conclusion also can be supported by other research that indicates sufficient depolymerization and devulcanization can result in the reduction of viscosity [25]. 

#### 3.3.2. The Results of Temperature Sweep Tests 

**Figure 3:**
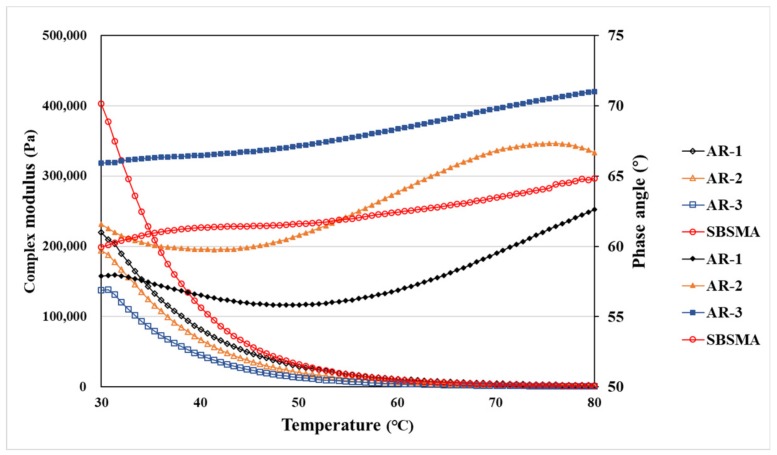
Results of temperature sweep.

The viscoelastic property of asphalt binders, including AR, vary greatly with temperature and is the main characteristic of rheology. The temperature sweep test was employed to measure the viscoelastic property from 30–80 °C. Complex modulus is an indicator of the stiffness and elastic component of materials. Occurring at high temperatures, the main challenge facing asphalt pavement is rutting, so a higher complex modulus is preferred due to its better anti-deformation performance. Conversely, the phase angle could indicate the viscous component of the materials, and a high phase angle (high viscous component) may indicate a poor anti-deformation. The rutting factor (G^*^/sinδ) combines the complex modulus and phase angle to evaluate the property of anti-rutting versus temperature. The data, including complex modulus, phase angle and rutting factor, are shown in Figure 3 and the high temperature PG grade of all samples is summarized in Table 6.

Considering Figure 3, an obvious gap exists between CR-1 and CR-3, and the curves of CR-2 remain in the middle of curves of CR-1 and CR-3 while the curves of SBS modified asphalt stayed above CR-1’s from 30 °C to about 55 °C. Subsequently, the figures for CR-1 overtook SBS modified asphalt’s. Regarding the phase angle, there is an upward trend for all samples, while the curves of CR-3 and CR-1 stay at the top and bottom, respectively. The curves of the rutting factors show a similar characteristic as the complex modulus’. 

Regarding Table 6, it is seen that the high-temperature performance of SBS modified asphalt and CR-2 are close, with the same PG grade of 82 °C, while the PG grades for CR-1 and CR-3 were lowest and highest at 76 °C and 88 °C, respectively. The results show that the addition of CR has a great effect on improving the high-temperature performance of binders because the PG grade of CR-1 is even higher than SBS modified asphalt. Using microwaves, however, can cause an adverse impact on the high-temperature performance, as seen when the PG grade of CR-3 decreased 12 °C compared with CR-1. To conclude, AR could be qualified to be used in high-temperature areas, while it should be noted the microwave processing has an effect of reducing the PG grade of AR binders. 

#### 3.3.3. High-Temperature Resistance to Permanent Deformation 

**Figure 4:**
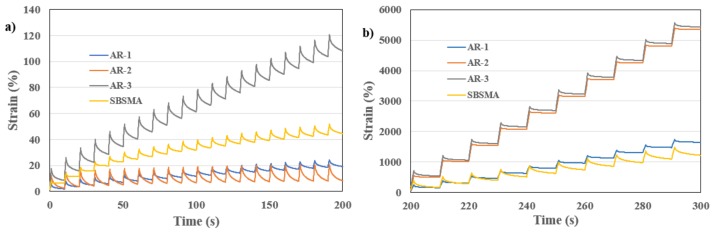
MSCR results at 64 °C. (**a**) load of 0.1 kPa; (**b**) load of 3.2 kPa.

Beyond the indicator of G^*^/sin*δ*, to investigate the resistance to permanent deformation at high temperature further, the MSCR was used. The anti-deformation properties depend on two factors: the stiffness, which decides the initial strain, and the strain recovery ability, which decides how much strain could be recovered and what final deformation could be left. The time–strain response of all samples curing the MSCR test are summarized in Figure 4, and the recoverable rate (R) and non-recoverable compliance (Jnr) are shown in Table 7.

The parameters R and Jnr could indicate high-temperature anti-rutting performance. It should be noted that a higher Jnr would indicate a higher susceptibility to rutting. Looking at Table 7, SBS-modified asphalt shows a good anti-deformation performance at both loads of 0.1 and 3.2 kPa (72% and 68% for R at 0.1 and 3.2 kPa, respectively), while for all AR binders there is an obvious change of R between loads of 0.1 and 3.2 kPa, indicating AR binders are sensitive to the load, especially for CR-2 with an R of 97% in 0.1 kPa but 9% in 3.2 kPa. This also can be supported by Figure 4a,b where the curves for CR-2 were at the bottom in 0.1 kPa but went up and stayed at the top at 3.2 kPa. To conclude, CR-1 and SBS-modified asphalt binders show better performance for anti-rutting and the desulphurization process, including microwave and long screw extrusion, have a negative impact on this property and make binders more sensitive to the load. It should be noted, however, that all three AR binders and SBSMA would perform well at high-temperatures for anti-rutting and could be classified at the highest level of “E” at 64 °C (Extremely Heavy Traffic “E” Grade, according AASHTO MP 19-10), because the Jnr at 3.2 kPa of ARs and the SBSMA are smaller than 0.5.

#### 3.3.4. Low-Temperature Performance 

**Figure 5:**
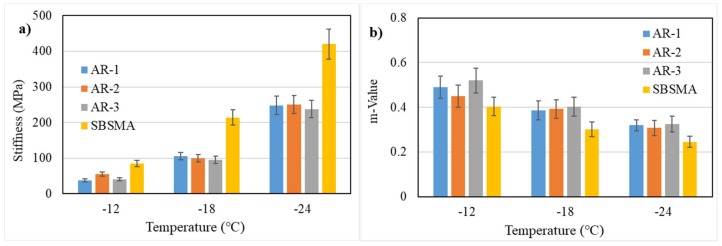
Stiffness and m-value in BBR tests. (**a**) stiffness; (**b**) m-Value.

Generally, the low-temperature performance of binders indicates anti-cracking properties, meanwhile the ductility at 5 °C cannot well reflect the low-temperature performance of AR binders because the particle effect of CR in AR binders cannot be ignored as it could have an effect of decreasing the ductility values. BBR was used to measure the low-performance of AR binders. The stiffness and m-value were thought to be well related to the low-performance of asphalt: the lesser stiffness and the bigger the m-value is, the better anti-cracking properties the asphalt binders have. The results of stiffness and m-value are summarized in Figure 5.

Viewing Figure 5, it is observed that all AR binders show a better low-temperature anti-cracking performance with lower stiffness values and higher m-value at each temperatures as compared to SBSMA, indicating the addition of CR into asphalt has an effect of improving the low-temperature of binders, even better than SBS-modified asphalts. It is seen that the low-temperature performance of CR-1and CR-3 were very close and even CR-3 performed better at −18 °C while the low-temperature performance of CR-2 was slightly inferior to CR-1 and CR-3. The low temperature grade of all three AR binders could reach −18°C. To conclude, microwave processing did not exert much adverse impact on low-temperature performance and all AR binders have a good low-temperature performance.

## 4. Conclusions

During this study, the microwave processing of CR was utilized to reduce the viscosity of AR binder systems. Desulphurization of CR was achieved by breaking S–C and S–S bonds without the C–C bonds by controlling the maximum power of the microwaves and the working time. Then, the binding performance of AR binders was evaluated regarding its storage stability, viscosity, high-temperature anti-rutting and low-temperature resistance to cracking. The main conclusions of this research are summarized below:

(1) The swelling results showed that the swelling rate of CR-3, processed by microwave, was two times as high as CR-1’s (without processing). Meanwhile, the sizes of CR-1 and CR-2 particles were close. Considering the extraction test results, the dissolving rate in the base binder of CR-3 increased more than two times as compared with CR-1, and the dissolution rate of CR-2 was also one time bigger than CR-1’s. So, the substance exchange between CR and the base asphalt could be accelerated by desulphurization, especially by microwave processing.
(2) The viscosity results showed that the viscosity of CR samples after desulphurization was consistently lower than CR-1’s at all temperatures and, especially, microwave processing had a noticeable effect of reducing viscosity (a viscosity decrease of 65% occurred at 190 °C). The storage stability result showed that the storage stability would be negatively affected by desulphurization, but it was still better than that of SBS-modified asphalt.
(3) The high-temperature performance results showed that the high temperature PG grade decreased from 88 °C to 76 °C and the Jnr parameters of 0.1 and 3.2 kPa increased more than fivefold and threefold, respectively, after microwave processing, indicating that the microwave processing CR would exert a negative impact on the high-temperature anti-rutting performance of AR binders. Additionally, BBR results showed that the stiffness and m-value decreased and increased slightly at all temperatures, respectively, after microwave processing, indicating the low-temperature anti-cracking performance is improved. Also, low-temperature results of all AR binder samples performed better than SBS modified asphalt.

## Figures and Tables

**Table 1:** Basic information of the base asphalt.

Items	Specifications	Measured Values
Penetration (25 °C, 0.1 mm)	ASTM D5	89.8
Penetration index PI	ASTM D5	−0.5
Softening point ( , °C)	ASTM D36	44.6
Ductility (15 °C, cm)	ASTM D113	165
Kinematic Viscosity (135 °C, Pa·s)	ASTM D4402	0.413
Density (15 °C, g/cm^3^)	ASTM D70	1.025

Where ASTM represents for American Society for Testing and Materials and R&B for the ring and ball method in softening point testing.

**Table 2:** The results of volume of CR particle in AR binders.

Samples	Measured Value (%)										Average Value (%)
CR-1	5.88	3.73	7.56	5.27	5.75	6.01	9.53	3.66	5.71	5.96	5.91
CR-2	8.23	3.8	9.84	6.18	5.92	5.02	4.93	5.12	4.17	4.01	5.72
CR-3	12.113	12.51	9.46	9.16	10.46	7.75	9.39	14.21	9.89	9.36	10.43

**Table 3:** Results of the dissolving rate in Toluene and base asphalt.

Samples	Toluene (%)	Base Asphalt (%)
CR-1	6.2	12.2
CR-2	5.0	27.0
CR-3	5.7	39.4

**Table 4:** Fundamental properties of AR and SBSMA binders.

Items	Specifications	Samples			
		AR-1	AR-2	AR-3	SBSMA
Penetration (15 °C, 0.1 mm)	ASTM D5	23.5	24.7	20.9	24.5
Penetration (25 °C, 0.1 mm)		55.4	58.3	52.8	58.6
Penetration (30 °C, 0.1 mm)		78.4	78.7	79.6	90.6
Penetration Index		0.8696	1.0906	0.1868	0.3627
Ductility (5 °C, cm)	ASTM D113	9.5	9.0	6.4	24.2
Softening point (°C)	ASTM D36	62.5	61.6	56.9	83.0
Elastic recovery rate (%)	ASTM D6084	62.03	65.23	59.81	62.03

**Table 5:** Results of storage stability tests.

Samples	Top (°C)	Bottom (°C)	Difference Value (°C)
AR-1	53.2	56.3	3.1
AR-2	52.2	58.1	5.9
AR-3	50.6	55.1	4.5
SBSMA	75.4	64.8	10.6

**Table 6:** High-temperature PG grades of AR and SBSMA binders.

Samples	G^*^/sin(*δ*) (kPa)						PG Grade (°C)
	58 °C	64 °C	70 °C	76 °C	82 °C	88 °C	
AR-1	16.2	9.3	5.6	3.4	2.2	1.01	88
AR-2	10.0	5.5	3.1	1.8	1.1	/	82
AR-3	6.0	3.3	1.9	1.1	0.7	/	82
SBSMA	14.2	7.4	4.1	2.5	1.3	/	82

**Table 7:** Recovery rate (R) and non-recoverable compliance (Jnr) at 64 °C.

Samples	0.1 kPa		3.2 kPa	
	R (%)	Jnr (×10^−3^)	R (%)	Jnr (×10^−3^)
AR-1	84.1	9.6	34.6	51
AR-2	96.7	4.1	8.9	167.1
AR-3	68.9	54.0	21.1	166.1
SBSMA	71.6	22.2	68.0	36.7

## References

[1] Presti D.L. (2013). Recycled tyre rubber modified bitumens for road asphalt mixtures: a literature review. Constr. Build. Mater..

[2] Mashaan N.S., Ali A.H., Karim M.R., Abdelaziz M. (2014). A review on using crumb rubber in reinforcement of asphalt pavement. Sci. World J..

[3] Heitzman M. (1992). Design and construction of asphalt paving materials with crumb-rubber modifier. Transport. Res. Rec..

[4] Xiao F.P., Amirkhanian S.N. HP-GPC Approach to evaluating laboratory prepared long-term aged rubberized asphalt binders. Proceedings of the Geohunan International Conference.

[5] Xiao F., Zhao P.E.W., Amirkhanian S.N. (2009). Fatigue behavior of rubberized asphalt concrete mixtures containing warm asphalt additives. Constr. Build. Mater..

[6] Kim H.H., Mithil M., Soon-Jae L., Moon-Sup L. (2018). Characterization of recycled crumb rubber modified binders containing wax warm additives. J. Traffic Transp. Eng..

[7] Li R., Wang C., Wang P., Pei J. (2016). Preparation of a novel flow improver and its viscosity-reducing effect on bitumen. FUEL.

[8] Zhang H., Chen Z., Xu G., Shi C. (2018). Evaluation of aging behaviors of asphalt binders through different rheological indices. Fuel.

[9] Putman B.J., Amirkhanian S.N. (2005). Rubberized asphalt mixtures: a novel approach to pavement noise reduction. Urban Transp..

[10] Singh D., Ashish P., Jagadeesh A. (2018). Influence of particle and interaction effects of different sizes of crumb rubber on rheological performance parameters of binders. J. Mater. Civ. Eng..

[11] Putman B.J., Amirkhanian S.N. (2010). Characterization of the interaction effect of crumb rubber modified binders using HP-GPC. J. Mater. Civ. Eng..

[12] Putman B.J., Amirkhanian S.N. Crumb rubber modification of binders: interaction and particle effects. Proceedings of the Asphalt Rubber 2006 Conference.

[13] Lougheed T.J., Papagiannakis A.T. (1996). Viscosity characteristics of rubber-modified asphalts. J. Mater. Civ. Eng..

[14] Frantzis P. (2004). Crumb rubber-bitumen interactions: diffusion of bitumen into rubber. J. Mater. Civ. Eng..

[15] Shatanawi K., Thodesen C., Amirkhanian S. (2008). Effects of crumb rubber variability on failure temperature of crumb rubber modified binders. Road Mater. Pavement..

[16] Bahia H.U., Davies R. (1994). Effect of crumb rubber modifiers (CRM) on performance-related properties of asphalt binders. Asphalt Paving Technol..

[17] Wang S., Wang Q., Li S. (2016). Thermooxidative aging mechanism of crumb-rubber-modified asphalt. J. Appl. Polym. Sci..

[18] Chipps J.F., Davison R.R., Glover C.J. (2001). A model for oxidative aging of rubber-modified asphalts and implications to performance analysis. Energy Fuels.

[19] Presti D.L., Izquierdo M.A., Carrión A.J.D.B. (2018). Towards storage-stable high-content recycled tyre rubber modified bitumen. Constr. Build. Mater..

[20] Zhang J., Fan Z., Hu D., Hu Z., Pei J., Kong W. (2018). Evaluation of asphalt–aggregate interaction based on the rheological properties. Int. J. Pavement Eng..

[21] Shen J., Amirkhanian S., Xiao F. (2006). High-pressure gel permeation chromatography of aging of recycled crumb rubber-modified binders with rejuvenating agents. Transp. Res. Rec..

[22] Shen J., Amirkhanian S., Xiao F., Tang B. (2009). Influence of surface area and size of crumb rubber on high temperature properties of crumb rubber modified binders. Constr. Build. Mater..

[23] Abdelrahman M., Carpenter S. (1999). Mechanism of interaction of asphalt cement with crumb rubber modifier. Transpor. Res. Rec..

[24] Sun D.Q., Li L.H. (2010). Factors Affecting the viscosity of crumb rubber-modified asphalt. Liq. Fuels Technol..

[25] Yu G.X., Li Z.M., Zhou X.L., Li C.L. (2011). Crumb rubber-modified asphalt: microwave treatment effects. Petrol. Sci. Technol..

[26] Mashaan N.S., Karim M.R. (2013). Investigating the rheological properties of crumb rubber modified bitumen and its correlation with temperature susceptibility. Mater. Res..

[27] Jeong K.D., Lee S.J., Amirkhanian S.N., Kim K.W. (2010). Interaction effects of crumb rubber modified asphalt binders. Constr. Build. Mater..

[28] Aflaki S., Memarzadeh M. (2011). Using two-way anova and hypothesis test in evaluating crumb rubber modification (CRM) agitation effects on rheological properties of bitumen. Constr. Build. Mater..

[29] Wang T., Xiao F., Amirkhanian S., Huang W., Zheng M. (2017). A review on low temperature performances of rubberized asphalt materials. Constr. Build. Mater..

[30] Lee S.J., Amirkhanian S.N. Effects of reaction time on physical and chemical properties of rubber-modified binders. Proceedings of the International Rubber Conference.

[31] Mashaan N.S., Ali A.H., Karim M.R., Abdelaziz M. (2011). Effect of blending time and crumb rubber content on properties of crumb rubber modified asphalt binder. Int. J. Phys. Sci..

[32] Zhu J., Birgisson B., Kringos N. (2014). Polymer modification of bitumen: advances and challenges. Eur. Polym. J..

